# Supplementary figures and images for: The metabolic pathways of carbon assimilation and polyhydroxyalkanoate production by Rhodospirillum rubrum in response to different atmospheric fermentation
Source: PLoS One. 2024 Jul 24;19(7):e0306222. doi: 10.1371/journal.pone.0306222 (PMC11268599; doi:10.1371/journal.pone.0306222)

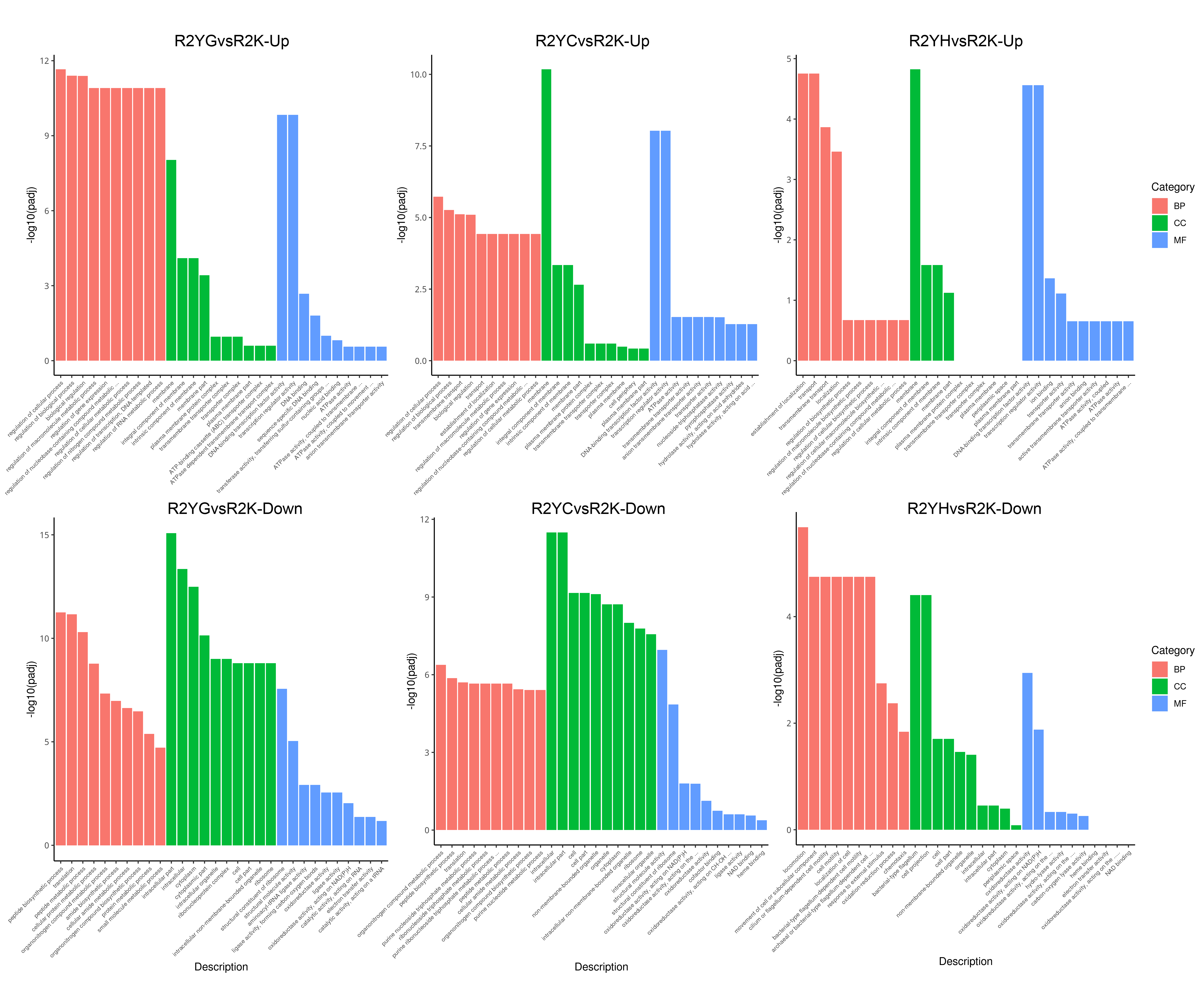

Supplement: S1 Fig — (TIF) [file pone.0306222.s001.tif]
