# Supplementary material for: The metabolic pathways of carbon assimilation and polyhydroxyalkanoate production by Rhodospirillum rubrum in response to different atmospheric fermentation
Source: PLoS One. 2024 Jul 24;19(7):e0306222. doi: 10.1371/journal.pone.0306222 (PMC11268599; doi:10.1371/journal.pone.0306222)
Supplement: Attachment — Submitted filename: Response to Reviewers.docx [file pone.0306222.s006.docx]

**Response to reviewers, PONE-D-24-01154**

Dear Editor,

We are grateful for the opportunity to revise our manuscript entitled “The metabolic pathways of carbon assimilation and polyhydroxyalkanoate production by Rhodospirillum rubrum in response to different atmospheric fermentation” in light of the editorial and reviewer comments. Below,

we have provided a point-by-point response.

Academic editor’s comments

**Reviewer #1:**

**Methods:**
1. GC analysis of PHA, How did the authors analysed the monomeric composition of PHA in GC analysis, did they performed Mass spectroscopy or they used the standard of 3-HV. Explain and mention in the section?
2. Mention the name of target genes for the qRT-PCR analysis in the section.
3. Give a brief methodology (in Methods section) regarding the fed-batch fermentation of the R. rubrum in a 3-L bioreactor.

**Response:**

We feel great thanks for your professional review work on our article. According to your nice suggestion, we have re-written the “materials and methods” part.

1. For PHA quantification, we used gas chromatography (GC) analysis. The standard curves of 3-HB and 3-HVwere generated to calculate PHBV content. And a commercial PHBV polymer (8 mol% PHV, Aladdin) were used to verify the methanolysis efficiency of PHBV. Meanwhile, the GC analysis was validated using crotonic acid and 2-pentenonic acid method by HPLC.

2. We have mentioned the name of thirteen target genes for the qRT-PCR analysis in the “Real-time qRT-PCR assays” section.

3. We have added a description of the fed-batch fermentation of the *R. rubrum* in a 3-L bioreactor in “Fed-batch experiments in bioreactor” section.

**Results and discussion:**
4. At 50% CO conditions, the bacterium uptake and utilization rates of CO and 3-HV accumulation were increased. Whereas, the genes which supply the precursor molecules such as propionyl-Co-A (genes in isoleucine degradation pathway in Fig. 6 i.e., Rru_A1946) are found to be less abundant in 50% CO condition. Explain and discuss.

**Response:**

We are grateful for your suggestion and have added more details of gene expression analysis. Samples of 8^th^ day gas fermentation were selected for RNA sequencing. And at this time point the cellular PHA formation in 50% CO fermentation might reach to platform stage. And with the shortage of carbon sources PHA would undergo a rapid mobilization. Therefore, those precursor molecules biosynthesis-related gene might show a downward trend. Additionally, as mentioned by the reviewer, *Rru_A1946* has indeed decreased in 50% CO condition. From this perspective, the content of 3-HV precursor molecule propionyl-Co-A would decrease, leading to a decrease in PHV content. However, the source of 3-HV precursor molecule was not limited to the isoleucine degradation pathway. The pyruvate synthesized by PFOR (pyruvate ferredoxin oxidoreductase) can also be converted to 3-HV precursor molecule propionyl-Co-A. And the PFOR coded gene *Rru_A2398*, were observed to be more abundant in 50% CO fermentation condition. These pathways collectively affect the content of propionyl-Co-A.

5. Rewrite the sentence “transcriptomic data and RT-PCR expression assay revealed that the expression level of RuBisCO showed no variation which implied the CBB pathway was not essential for CO2 fixation under 50% CO gas atmosphere (Fig.5)”. The sentence sounds confusing, mention the substrate conditions used for the comparison.

**Response:**

We apologize for the language problems in the original manuscript. We have corrected the sentence into “The transcriptomic data and RT-PCR expression assay both revealed that the expression level of *RuBisCO* showed no variation between growth phase and 50% CO fermentation phase, which implied the CBB pathway was not essential for CO_2_ fixation under 50% CO gas atmosphere (Fig 5).”

6. Clearly discuss, whether R. rubrum adopt photoautotrophic mode or chemoautotrophic mode of nutrition in the syngas conditions (when hydrogen was added).

**Response:**

Thank you for your suggestion and we have added the table 4. In the table, we have clearly pointed out *R. rubrum* adopted photoheterotrophic mode of nutrition in the syngas condition.

7. The fed batch process was performed with CO:H2 ratio at 3:1 and feeding the acetate. Why did author add 10 mM of acetate on 4th day of CO/H2 cultivation whereas acetate was added on 7th day of pure CO condition?

**Response:**

As reviewer suggested，we have added more details of acetate feeding experiment. Analysis of acetate content shown that the initial H_2_ addition excited acetate utilization. On the 4^th^ day of CO/H_2_ cultivation, the initial residual acetate amount was only 31%, but for pure CO fermentation until to the 7^th^ day the initial residual acetate amount was decreased to 29%. Therefore, we added 10 mM acetate on 4^th^ day of CO/H_2_ cultivation and on the 7^th^ day of pure CO condition to re-started PHA formation.

8. What is the monomeric composition of the PHA accumulated in CO:H2 ratio at 3:1 and 10 mM addition of acetate? Author have used the term PHA in this section and if the PHA monomer were analysed, author should discuss here.

**Response:**

Thank you for your suggestion and we have added the table 3. In the table, we have shown with 10 mM acetate feeding the monomeric composition of the PHBV accumulated in CO:H_2_ ratio at 3:1 (6.2 % mol 3HV) and in pure CO (2.4% mol 3HV).

9. Author should compare and discuss the fed batch results of R. rubrum using syngas to PHA production in the light of some relevant literature.

**Response:**

We think this is an excellent suggestion. We have added a comparison table (table 4) in comparison with the results of previous studies on PHA production. According to table 4, our procedures indeed increased PHA production when compared to previous works operating in the same culture medium. Our pure CO fermentation results were very close to those achieved with *R. rubrum* on syngas in continuous light or dark cultures. Furthermore, our PHBV productivity in syngas atmosphere is better than others achieved with syngas or CO fermentation and achieve its maximum PHBV yield in 6 days. Therefore, it is interesting for further developing new strategies for initial syngas selection and gas composition regulation to improve PHA production of *R. rubrum*.

10. Conclusions: Concise the conclusion section, and shift the statements of results and discussion to relevant section.

**Response:**

Thank you for your suggestion and we have concisely re-written the conclusion section and shifted the statements of results and discussion to relevant section.

**Minor comments**
11. Change the Rs. Rubrum to R. rubrum in the abstract and elsewhere.
12. What is RRNCO medium? Provide the details.
13. Use a consistent style of writing units, for example ΜL should be ml or mL, hour or h ?
14. This seems to be contradicting results, author should explain ?
15. Figure 1 and Fig. 7, the figure markers are not clear in the figures
16. Why did authors used different style of writing the heading in materials and methods section (e.g, first letter capital in “Monitoring of the Acetate Consumption” whereas some heading are in sentence case. Use a similar style.
17. Take care of spacing between the words throughout the manuscript.
18. Mention the source of the commercial kits used in the study, for example cDNA synthesis kit and library preparation kits etc.
19. Italicize the scientific name in the references and elsewhere.

**Response:**

We sincerely thank you for careful reading. As your suggestions, we have corrected the Rs. Rubrum into R. rubrum in the abstract and elsewhere.

The details of RRNCO medium have been provided in the “materials and methods” part.

We have reviewed the whole paper and used a consistent style to rewrite the units (e.g. ml, h).

For the contradicting results, we have tried our best to provide reasonable explanations.

The figure markers of figure 1 and 7 have been re marked.

We have standardized the heading style in materials and methods section and changed “Monitoring of the Acetate Consumption” into “Acetate content analysis”.

We have checked the whole paper and modified the spacing between the words.

We have supplemented the source of the commercial kits used in this study.

We have checked the whole paper and italicized the scientific name.

**Reviewer #2:**

1. In the introduction section, there are more complete and current articles at shaken flasks or bioreactor scale that describe and analyze the influence of the CO fraction on the production of H_2_ and the accumulation of PHB with this microorganism (see Rodríguez et al., 2021. Biotechnology for Biofuels 14: 168 and Godoy et al 2024. Applied Microbiol. Biotechnol. 108(1), 258). These works must be included as references in the text.

**Response:**

We feel great thanks for your professional review work on our article. According to your nice suggestion, we have added those current articles on “at shaken flasks or bioreactor scale that describe and analyze the influence of the CO fraction on the production of H_2_ and the accumulation of PHB with this microorganism (Rodríguez et al., 2021 and Godoy et al 2024)” as references into the Introduction part in the revised manuscript.

2. Materials and methods: in bacterial strain, growth and media subsection, authors must clear indicate that the microorganism was grown under photoheterotrophic conditions. Additionally, the characteristics of the UV lamp used in the light shaker should be included in this section.

**Response:**

We are grateful for your suggestion and have added more details of bacterial strain, growth and media subsection. We have clearly indicated the two-step culture strategy used in this paper for PHA accumulation in *R. rubrum*. First, an enriched broth medium was used for rapid microbial heterotrophic growth. Then a modified RRNCO medium was adopted for subsequent microbial anaerobic photoheterotrophic cultivation to accumulate PHA. And 30 μE white lights were used for photoheterotrophic cultivation, UV lamp was not used in the entire cultivation process.

3. PHB extraction and quantification by GC analysis: Authors mention that a calibration curve has been used to quantify the intracellular content of the biopolymer. However, methanolysis with PHBV has a low yield (around 20% of methylesthers detected by GC/MS with respect to the initial amount treated in the calibration curve). How was the calibration curve made? Have the authors used PHB or PHBV? Has this low extraction yield been taken into account? Authors should clarify and expand on it in this section. Otherwise, NMR analysis is more accurate to determine the molar monomeric distribution of the biopolymer.

**Response:**

We sincerely thank you for careful reading. As your suggestions, we have re-written the “PHA extraction and quantification” in Materials and methods part. For PHA quantification, we used gas chromatography (GC) analysis. The standard curves of 3-HB and 3-HVwere generated to calculate PHBV content. And a commercial PHBV polymer (8 mol% PHV, Aladdin) were used to verify the methanolysis efficiency of PHBV. Meanwhile, the GC analysis was validated using crotonic acid and 2-pentenonic acid method by HPLC. As mentioned by the reviewer, NMR analysis is indeed more accurate to determine the molar monomeric distribution of the biopolymer. NMR analysis has been used in our previous research for PHA fructose fermentation (Liu et al., 2019). Compared with NMR results, gas chromatography analysis could correctly detect the molar monomeric distribution of the biopolymer. Moreover, because our laboratory is not equipped with the relevant instruments for NMR analysis, we are very sorry that we cannot supplement this relevant experiment in this research.

4. Result section: In Fig. 1 authors explained that fructose was used as a sole carbon source in preliminary cultures, but in text they wrote "the bacteria were firstly transferred to (...) medium containing glucose or CO as...". If they have feed fructose, it should be clear in the text. Moreover, in Fig 1.A, fructose profile must be included in order to facilitate the understanding of experimental results. In Fig 1C, legend of condition 100%CO was the same as 20%CO. Authors should unify the format of this figure in the final version of the manuscript.

**Response:**

We were really sorry for our careless mistakes. Thank you for your reminder. Fructose was indeed used as a sole carbon source for PHA inducing cultivation. We have corrected the sentence into “The bacteria were firstly cultured in growth medium 3 days to achieve a high cell density, then transferred to the induced medium containing fructose or CO as the sole source of carbon and energy”. As reviewer suggested, we have added fructose profile to facilitate the understanding of fructose fermentation experimental results. And the legends of condition 100%CO and 20%CO in Fig 1C have been re marked.

5. Result section "fed-batch cultivation with R. rubrum on syngas". It is necessary a better explanation about how the experiment was carried out in the bioreactor and a brief description of the equipment. (for example, the temperature is not specified, nor the gas flow rate used, nor how the proportion of H_2_/CO fed is regulated). Moreover, in the text it is not clear the use of acetate, or why. On the other hand, I believe that these explanations should be included in the materials and methods section, where authors must mention the information included in Figure 8.A. Finally, what fraction of the outlet gas stream returns to the broth according to the scheme shown in Fig 8.A?

**Response:**

We are grateful for your suggestion. The description of “fed-batch cultivation with *R. rubrum* in bioreactor” should be include in the materials and methods section. We have added a detailed description of the “Fed-batch experiments in bioreactor” in the materials and methods part, including the fermentation equipment, temperature, gas flow rate, how the proportion of H_2_/CO fed is regulated, the acetate supplement strategy, what fraction of the outlet gas stream returns to the broth, etc). For the use of acetate, as reported by Revelles et al that acetate providing the carbon skeleton for PHAs and promoting PHA accumulation. Consistently, our research shown acetate feeding could promote the cellular PHA formation to a higher yield. We have added these explanations in the result section.

6. Result section "fed-batch cultivation with R. rubrum on syngas": Why the authors started the fermentations with 2 g/l of initial biomass? Have they used the same RRNCO medium or the bioreactor runs were performed as a resting cell bioprocess? The evolution of biomass profiles in Fig. 8 suggests this idea, but authors have not clarify or mention in the text. This section must be highly improved. To enhance the understanding of the evolution of the experiments, the acetate concentration profiles in the runs carried out with pure CO and CO/H_2_ 75/25 should be included in Figures 8E and 8F, respectively. Figure 8D does not provide any relevant information in this study. I suggest that the most powerful results obtained in the bioreactor runs must be included in a Table, in order to see the effect of H2 over biopolymer production and to compare with other studies available in the literature. On the other hand, why has the monomeric distribution of the PHA produced in the bioreactor was not been included in Fig 8, if it is the most important object of study in this article?

**Response:**

We sincerely appreciate the valuable comments. To clearly descript the results of "fed-batch cultivation with R. rubrum on syngas" section, we first have added more details of “Bacterial strain, growth and media subsection” and “Fed-batch experiments in bioreactor” in the materials and methods part. As mentioned in materials and methods section, we used the same RRNCO medium and stationary phase cells for fed-batch experiments. Specifically, 3-L stationary phase grown pre-culture were centrifuged and resuspended with 0.6-L RRNCO medium in bioreactor for gas fermentation and the initial biomass was around 2 g/l. Moreover, in the resting cells polymer accumulation prevailed over residual biomass formation. We have re-written the relevant information in the result section. To enhance the understanding of the evolution of the experiments, as mentioned by the reviewer, the changes of acetate concentration profiles in pure CO and CO/H_2_ have been added Figures 8E and 8F, respectively. In order to see the effect of H_2_ over biopolymer production, as mentioned by the reviewer, we have added the table 3, in which the most powerful results obtained in the bioreactor runs were included: with 10 mM acetate feeding the yield of the PHBV reaching to 20 % ww^-1^ (6.2 % mol 3HV) on the 6^th^ day of CO/H_2_ cultivation and 28% ww^-1^ (2.4 % mol 3HV) on the 12^th^ day of pure CO cultivation. The monomeric distribution of the PHA produced in the bioreactor have been included in table 3. Furthermore, we have added a comparison table (table 4) in comparison with the results of previous studies on PHA production. According to table 4, our procedures indeed increased PHA production when compared to previous works operating in the same culture medium. Our pure CO fermentation results were very close to those achieved with *R. rubrum* on syngas in continuous light or dark cultures. PHBV productivity in syngas atmosphere is better than others achieved with syngas or CO fermentation and achieve its maximum PHBV yield in 6 days. Therefore, it is interesting for further developing new strategies for initial syngas selection and gas composition regulation to improve PHA production of *R. rubrum*.

7. Finally, the quality of the figures in general must be improved. In the documentation available for revision, they appear very pixelated, so that it is difficult to read the axes of the figures and the text that appears in them.

**Response:**

Thank you for your suggestion and we have provided high quality figures in the final version of the manuscript to ensure clear visibility of the axes of the figures and the text that appears in them.

**Reviewer #3:**

1. A comparison table should be provided that shows the growth of the strain and PHA productivity from Rhodospirillum rubrum using CO_2_ or CO, in comparison with the results of previous studies on PHA production.

**Response:**

We feel great thanks for your professional review work on our article. According to your nice suggestion, we have added the table 3, in which the most powerful results obtained in the bioreactor runs using CO and syngas (75%CO and 25%H_2_) were included: with 10 mM acetate feeding the yield of the PHBV reaching to 20 % ww^-1^ (6.2 % mol 3HV) on the 6^th^ day of CO/H_2_ cultivation and 28% ww^-1^ (2.4 % mol 3HV) on the 12^th^ day of pure CO cultivation. Furthermore, we have added a comparison table (table 4) in comparison with the results of previous studies on PHA production. According to table 4, our procedures indeed increased PHA production when compared to previous works operating in the same culture medium. We have re-written the relevant information in the result section.

2. It's necessary to investigate or provide opinions on whether the addition of hydrogen affects only CO_2_ conversion or if there are other aspects that it might influence within the PHA biosynthesis pathway.

**Response:**

We are grateful for your suggestion and have added more details of short-chain-length PHAs (monomer units of C3 to C5) biosynthesis pathway. Syngas (50%CO and 50%H_2_) fermentation and pure CO fermentation uses the same carbon source substrate. PHAs are both synthesized from acetyl-CoA via three enzymatic reactions by three distinct enzymes. In our opinions, the addition of hydrogen activated different CO_2_ assimilation and redox balancing route. The various intermediate metabolites of these pathways then, composed different precursor molecules for PHA synthesis. The addition of hydrogen promoted the synthesis of 3-HV precursor molecule propionyl-Co-A and finally leading to increase in HV content. In term of PHA yield, the addition of hydrogen activated the BCAA biosynthesis pathway which act as a key electron-sinking pathway to bypass PHA production, and then eventually lead to the decrease of intracellular PHA accumulation.

3. There should be a detailed examination and comparison with previous research results on the PHA productivity per unit time and the conversion rate of the supplied carbon. Based on the results presented now, the PHA productivity appears to be very low compared to when other carbon sources were used, making it difficult to gauge the competitiveness in terms of cost-effectiveness.

**Response:**

We sincerely appreciate the valuable comments. We have we have added the table 3, in which we descripted the consumption rate of the supplied carbon (CO and acetate) and the yield of PHBV. Furthermore, we have added a comparison table (table 4) in comparison with the results of previous studies on PHA production. According to table 4, our pure CO fermentation results were very close to those achieved with *R. rubrum* on syngas in continuous light or dark cultures. PHBV productivity in syngas atmosphere is better than others achieved with syngas or CO fermentation and achieve its maximum PHBV yield in 6 days. Therefore, it is interesting for further developing new strategies for initial syngas selection and gas composition regulation to improve PHA production of *R. rubrum*.
