# Supplementary material for: The metabolic pathways of carbon assimilation and polyhydroxyalkanoate production by Rhodospirillum rubrum in response to different atmospheric fermentation
Source: PLoS One. 2024 Jul 24;19(7):e0306222. doi: 10.1371/journal.pone.0306222 (PMC11268599; doi:10.1371/journal.pone.0306222)
Supplement: Attachment — Submitted filename: Response to Reviewers.docx [file pone.0306222.s007.docx]

**Response to reviewers, PONE-D-24-01154**

Dear Editor,

We are grateful for the opportunity to revise our manuscript entitled “The metabolic pathways of carbon assimilation and polyhydroxyalkanoate production by Rhodospirillum rubrum in response to different atmospheric fermentation” in light of the editorial and reviewer comments. Below,

we have provided a point-by-point response.

**Journal Requirements:**

Please review your reference list to ensure that it is complete and correct. If you have cited papers that have been retracted, please include the rationale for doing so in the manuscript text, or remove these references and replace them with relevant current references. Any changes to the reference list should be mentioned in the rebuttal letter that accompanies your revised manuscript. If you need to cite a retracted article, indicate the article’s retracted status in the References list and also include a citation and full reference for the retraction notice.

**Response:**

We have reviewed our reference list and ensured that it was complete and correct. There were no changes to the reference list.

**Academic editor’s comments**
1. The newly added table 4 can be presented in concise manner. I suggest to merge the column with similar informations and reference.

**Response:**

We feel great thanks for your professional review work on our article. According to your nice suggestion, we have merged the column with similar information (growth conditions, cultivation modes) and reference in table 4. It does look more concise now.

2. Abbreviate the polyhydroxybutyrate (PHB) and poly(3-hydroxybutyrate-co-3-hydroxyvalerate) (PHBV) should be mentioned at the place of their first appearance in the text.

**Response:**

Thanks for your careful checks. We have added the abbreviations of the polyhydroxybutyrate (PHB) and poly(3-hydroxybutyrate-co-3-hydroxyvalerate) (PHBV) at their first appearance in the text.

3. Author can improve the resolution of images, for example, Figure 3A, description on x-axis are not clearly visible.

**Response:**

Thank you for your suggestion and we have provided high quality figures in the final version of the manuscript to ensure clear visibility of the axes of the figures and the text that appears in them. But，we are sorry that during the image upload process, the system seems to automatically compress the image quality. We have resubmitted the figure 3.

4. Thoroughly check the manuscript for typographical errors (Example, R.rubrum mentioned in abstract and keywords, put a space in between R. and rubrum, it should be R. rubrum).

**Response:**

We sincerely thank you for careful reading. As your suggestions, we have thoroughly checked the manuscript for typographical errors and corrected the *R.rubrum* into *R. rubrum* in the abstract, keywords and elsewhere.

5. Italicise the Rhodospirillum rubrum in the legends of table 3 and 4 and elsewhere (if any).

**Response:**

We feel sorry for our carelessness. In our resubmitted manuscript, we have italicised the *Rhodospirillum rubrum* in the legends of table 3 and 4 and elsewhere.

6. Make a uniformity in writing the units as per the style of journal (example, g L-1 or g/L in table 3, table 4 and in the text elsewhere. Another example is, author are using ml for millilitre and L for litre).

**Response:**

Thank you for your kind suggestion. We have reviewed the whole paper and used a consistent style to rewrite the units. (for example, changing g/L into g l^-1^ in table3, 4 and anywhere, using l for litre)
